# Biocompatible combinations of nisin and licorice polyphenols exert synergistic bactericidal effects against *Enterococcus faecalis* and inhibit NF-κB activation in monocytes

**DOI:** 10.1186/s13568-020-01056-w

**Published:** 2020-07-06

**Authors:** Daniel Grenier, Eve Marcoux, Jabrane Azelmat, Amel Ben Lagha, Philippe Gauthier

**Affiliations:** Oral Ecology Research Group, Faculty of Dentistry, Université Laval, 2420 de la Terrasse, Quebec City, QC G1V 0A6 Canada

**Keywords:** *Enterococcus faecalis*, Nisin, Licorice, Polyphenols, Biofilm, Synergy, NF-κB, Monocytes

## Abstract

*Enterococcus faecalis* is one of the bacterial species most frequently isolated from persistent endodontic and apical periodontal infections. The aim of the present study was to evaluate the synergistic antibacterial effects of nisin and selected licorice polyphenols (glabridin, licoricidin, licochalcone A) against planktonic and biofilm-embedded *E. faecalis* cells. The biocompatibility and anti-inflammatory properties of the nisin/licorice polyphenol combinations were also investigated. The lantibiotic bacteriocin (nisin), the two isoflavonoids (glabridin, licoricidin), and the chalcone (licochalcone A) efficiently inhibited the growth of *E. faecalis*, with MICs ranging from 6.25 to 25 µg/mL. Combining nisin with each licorice polyphenol individually resulted in a significant synergistic antibacterial effect. Following a 30-min contact, nisin in combination with either glabridin, licoricidin, or licochalcone A caused significant biofilm killing. The nisin/licorice polyphenol combinations had no cytotoxic effects (oral epithelial cells, gingival fibroblasts, and stem cells of the apical papilla), with the exception of nisin/glabridin, when used at their MICs. Lastly, we showed that nisin/glabridin, nisin/licoricidin, and nisin/licochalcone A inhibit NF-κB activation induced by *E. faecalis* in a monocyte model, suggesting that these combinations possess anti-inflammatory properties. The present study provides evidence that combinations of nisin and glabridin, licoricidin, or licochalcone A show promise as root canal disinfection agents.

## Key points

- Nisin and licorice polyphenols exert synergistic antibacterial activity.
- Nisin and licorice polyphenols did not show any cytotoxicity on different oral cell types.
- Combinations of nisin and licorice polyphenols possess anti-inflammatory properties.

## Introduction

Although many microorganisms colonizing the oral cavity can penetrate and infect the dental pulp, only a limited number of species are truly capable of doing so given the particular environment of the root canal (Siqueira and Rocas 2009). Once they gain access to the complex root canal system, bacteria adhere to the dentine surface and form a biofilm, which is a complex aggregation of microorganisms entrapped in a polymer network mainly composed of polysaccharides and DNA. *Enterococcus faecalis* is one of the bacterial species most frequently isolated from persistent endodontic and apical periodontal infections (Roças et al. 2004; Sedgley et al. 2006). The exact source of this Gram-positive facultative anaerobic bacterium is not clear, although there is evidence that *E. faecalis* may have an exogenous origin rather than being a permanent resident of the oral cavity (Vidana et al. 2011). *E. faecalis* has the ability to form a dense biofilm, which makes this species much more resistant to host immune defenses as well as to antimicrobial agents used for root canal disinfection (Duggan and Sedgley 2007).

An important aspect of endodontic therapy is the use of irrigating solutions possessing antimicrobial activity against both planktonic and biofilm-embedded microorganisms. Endodontic treatments are aimed at eliminating the microorganisms in the root canal. However, given the anatomic complexity of the root canal system, this can be a challenge. If the endodontic treatment fails, the residual bacteria and their toxins may affect the periradicular tissues and create an inflammatory response resulting in apical periodontitis, which is characterized by the recruitment of inflammatory cells, including polymorphonuclear neutrophils and monocytes (Gomes and Herrera 2018). When the NF-kB signaling pathway is activated by bacteria or their cell surface components such as lipopolysaccharide (LPS) and lipoteichoic acid, these cells release pro-inflammatory mediators that contribute to tissue destruction (Gomes and Herrera 2018).

Sodium hypochlorite is one of the most commonly used root canal irrigating agents as it can dissolve organic matter and is a strong disinfectant (Goncalves et al. 2016). However, the extrusion of sodium hypochlorite beyond the apical foramen or through a perforation can lead to complications, from minor to severe tissue damage (De Sermeno et al. 2009). Because of this, research aimed at identifying novel non-cytotoxic irrigating agents with broad-spectrum antimicrobial activity is of great interest. Antimicrobial peptides and plant polyphenols may be promising candidates in this regard.

Nisin is a broad-spectrum bacteriocin naturally produced by *Lactococcus lactis* that has been extensively studied in the food industry (Hansen 1994). This antimicrobial peptide (34 amino acid residues) is currently approved for use as a food preservative in 48 countries. Nisin is particularly effective in killing Gram-positive bacteria by interacting with their negatively charged membranes, leading to pore formation (Lubelski et al. 2008). Polyphenols are plant secondary metabolites and are classified into different families based on their chemical structures (Fraga et al. 2019). Previous investigations have shown that several classes of polyphenols exhibit a broad spectrum of antimicrobial activity (Daglia 2012). More specifically, licorice-derived chalcones and isoflavonoids have been reported to possess marked antibacterial activity against both Gram-positive and Gram-negative bacteria (Messier et al. 2012).

The aim of the present study was to evaluate the synergistic antibacterial effects of nisin and selected licorice polyphenols (glabridin, licoricidin, licochalcone A) against planktonic and biofilm-embedded *E. faecalis* cells. The biocompatibility and anti-inflammatory properties of the nisin/licorice polyphenol combinations were also investigated.

## Materials and methods

### Compounds

Glabridin (Wako Chemicals, Richmond, VA, USA) and licochalcone A (Enzo Life Sciences, Inc., Farmingdale, NY, USA) were prepared in 95% (v/v) ethanol, while licoricidin (EMMX Biotechnology, Lake Forest, CA, USA) was prepared in dimethyl sulfoxide. Stock solutions (20 mg/mL) were kept in the dark at 4 **°**C for up to 1 month. Given that these stock solutions were highly diluted in the assays described below, neither ethanol nor dimethyl sulfoxide caused any biological effects (data not shown). Nisin A (Sigma-Aldrich Canada Co., Oakville, ON, Canada) was prepared in 0.02 N HCl at a concentration of 750 μg/mL and was sterilized by filtration through a 0.22-μm pore size membrane filter. Chlorhexidine digluconate (Sigma-Aldrich Canada Co.,), which was used as a positive antimicrobial control, was prepared in distilled water at a concentration of 5 mg/mL and was sterilized by filtration through a 0.22-μm pore size membrane filter.

### Bacteria and growth conditions

The reference strain *E. faecalis* ATCC 19433 as well as two clinical isolates of *E. faecalis* (0DOT, and 1DOT) were used. They were grown in Brain Heart Infusion broth (BHI; BBL Microbiology Systems, Mississauga, ON, Canada) supplemented with 0.5% glucose at 37 °C in an anaerobic chamber (80% N_2_/10% H_2_/10% CO_2_).

### Determination of minimum inhibitory and minimum bactericidal concentrations

The minimum inhibitory concentration (MIC) and minimum bactericidal concentration (MBC) values of nisin and the licorice polyphenols were assessed using the microdilution method as described by the Clinical and Laboratory Standards Institute (Wayne 2012). The bacterial suspension and two-fold serial dilutions of the compounds (from 200 µg/mL) diluted in culture medium were added to the wells of a 96-well microplate. Wells with no compounds (only carrier solvent) were used as controls (100% growth). After a 24-h incubation, microbial growth was assessed by recording the optical density at 660 nm (OD_660_) using a Synergy 2 microplate reader (BioTek Instruments, Winooski, VT, USA). The MIC values are the lowest concentrations of the compounds that completely inhibited bacterial growth. To determine the MBC values, 5-μL aliquots from the wells with no visible growth were spread on BHI agar plates, which were incubated for 3 days at 37 °C. The MBC values are the lowest concentrations at which no colony formation occurred. Assays were performed in triplicate in two independent experiments to ensure reproducibility. A representative set of data is presented.

### Biofilm formation

*E. faecalis* ATCC 19433 was grown (24 h) in the wells of a flat-bottomed 96-well microplate as described above in the absence (control) and presence of either nisin or licorice polyphenols at sub-inhibitory concentrations corresponding to 1/4 MIC. The medium, free-floating bacteria, and loosely-bound biofilm were then removed by aspiration, and the wells were gently washed three times with 50 mM phosphate-buffered saline (pH 7.2; PBS). The biofilms were stained with 0.04% crystal violet (100 µL) for 10 min. The wells were washed three times with PBS to remove unbound crystal violet dye and were dried for 2 h at 37 °C. After adding 100 µL of 95% (v/v) ethanol to each well, the microplate was shaken for 10 min to release the dye from the biofilms, and the absorbance at 550 nm (A_550_) was recorded using a Synergy 2 microplate reader. Assays were performed in triplicate in two independent experiments, and the means ± standard deviations were calculated.

### Synergistic antibacterial interactions

The synergistic antibacterial effects against *E. faecalis* ATCC 19433 of nisin in combination with the licorice polyphenols were evaluated using the checkerboard method (Eliopoulos and Moellering 1996). Compound A was serially diluted in culture medium (100 µL) along the ordinate of a 96-well microplate, while compound B was serially diluted in culture medium (100 µL) along the abscissa. A bacterial suspension prepared in fresh culture medium and adjusted to a McFarland standard of 0.5 was used as an inoculum. The microplate wells were inoculated with 100 µL of the suspension, and the microplate was incubated at 37 °C for 24 h in an anaerobic chamber. Wells with no bacteria or no compounds were included in the assay. After the incubation period, bacterial growth was assessed by recording the OD_660_ using a Synergy 2 microplate reader. The lowest concentration at which no growth occurred was considered the MIC. The fractional inhibitory concentration index (FICI) was calculated using the following equation: FICI = (MIC_A+B_/MIC_A_) + (MIC_B+A_/MIC_B_). An FICI ≤ 0.5 was considered as indicating a synergistic effect, an FICI > 0.5 and ≤ 4.0 as indicating no effect, and an FICI > 4.0 as indicating an antagonistic effect. Assays were performed in triplicate to ensure reproducibility. A representative set of data is presented.

### Biofilm killing

The ability of nisin/glabridin, nisin/licoricidin, and nisin/licochalcone A to kill *E. faecalis* ATCC 19433 biofilms was investigated. Briefly, 24-h biofilms were pre-formed in the wells of a 96-well microplate, washed once with PBS, and treated for 30 min with the above combinations. Each compound of a combination was used at a concentration corresponding to its MIC value (in PBS). Following these treatments, the biofilms were washed once with PBS. A series of biofilms was stained with 0.04% crystal violet as described above to determine biofilm desorption. A second series of biofilms was used to determine bacterial viability using a commercial luminescence assay (BacTiter-Glo™; Madison, WI, USA) that measures adenosine triphosphate (ATP), an indicator of metabolically active viable bacteria. Luminescence was quantified using a Synergy 2 microplate reader. All the assays were performed in triplicate in two independent experiments, and the means ± standard deviations were calculated.

### In vitro biocompatibility assay

The human oral epithelial cell line B11, which has already been characterized (Groeger et al. 2008), was kindly provided by S. Groeger (Justus Liebig University Giessen, Germany). Cells were cultured in keratinocyte serum-free medium (K-SFM; Life Technologies Inc., Burlington, ON, Canada) supplemented with growth factors (50 µg/mL of bovine pituitary extract and 5 ng/mL of human epidermal growth factor) and 100 µg/mL of penicillin G-streptomycin. The primary human gingival fibroblast cell line HGF-1 (CRL-2014) was purchased from the American Type Culture Collection (ATCC, Manassas, VA, USA) and was cultured in Dulbecco’s Modified Eagle’s Medium (DMEM; Life Technologies Inc.) supplemented with 4 mM l-glutamine, 15% heat-inactivated fetal bovine serum (FBS; HyClone Laboratories, Logan, UT, USA), and 100 µg/mL of penicillin G-streptomycin. The previously characterized human SCAP cell line (RP-89) was cultured in -minimum essential medium (MEM; Life Technologies Inc.) supplemented with 10% FBS, 2 mmol/L l-glutamine, and 100 µg/mL of penicillin G/streptomycin (Ruparel et al. 2013). All cell cultures were incubated at 37 °C in a 5% CO_2_ atmosphere. To evaluate the effect of nisin/glabridin, nisin/licoricidin, and nisin/licochalcone A on cell viability, cells were seeded (1 × 10^5^ cells in 100 µL) in the wells of a 96-well tissue culture plate and were incubated at 37 °C in a 5% CO_2_ atmosphere until they reached confluence. The cells were treated with the combinations for 2 h. Each compound of a combination was used at concentrations corresponding to the MIC and twofold MIC values obtained against *E. faecalis* ATCC 19433. Thereafter, an MTT (3-[4, 5-diethylthiazol-2-yl]-2,5-diphenyltetrazolium bromide) assay was performed according to the manufacturer’s protocol (Roche Diagnostics, Mannheim, Germany) to assess cell viability. The assays were performed in triplicate in two independent experiments, and the means ± standard deviations were calculated.

### NF-κB activation assay

The human monoblastic leukemia cell line U937-3xκB-LUC was kindly provided by R. Blomhoff (University of Oslo, Norway). It consists of the U937 cell line stably transfected with a luciferase gene coupled to a promoter of three NF-κB binding sites (Carlsen et al. 2002). Monocytes were grown at 37 °C in a 5% CO_2_ atmosphere in RPMI-1640 medium (Life Technologies Inc.) supplemented with 10% heat-inactivated FBS, 100 µg/mL of penicillin–streptomycin, and 75 µg/mL of hygromycin B. In a first analysis, the ability of *E. faecalis* ATCC 19433 to induce NF-κB activation was assessed. Briefly, 100 µL of the monocyte suspension (10^7^ cells/mL) was seeded in the wells of a black bottom, black wall 96-well microplate (Greiner Bio-One North America Inc., Monroe, NC, USA), and an overnight culture of *E. faecalis* suspended in culture medium was added at a multiplicity of infection (MOI) of 100, 50, 25, 12, 6, or 3. The plate was incubated at 37 °C (5% CO_2_) for a further 6 h. NF-κB activation was then assessed using the Bright-Glo™ Luciferase Assay System (Promega, Madison, WI, USA) by adding 100 µL of luciferase substrate to the wells at room temperature. Luminescence was recorded using the luminometer option of the Synergy 2 microplate reader (BioTek Instruments) within 3 min of adding the substrate. To investigate the ability of nisin/glabridin, nisin/licoricidin, and nisin/licochalcone A (each compound at their MIC, ½ MIC, and ¼ MIC obtained against *E. faecalis* ATCC 19433) to inhibit NF-κB activation, the combinations were added 30 min prior to challenge U937-3xκB-LUC cells with *E. faecalis* at an MOI of 100. All the assays were carried out in triplicate in two independent experiments, and the means ± standard deviations were calculated.

### Statistical analysis

Statistical analyses were performed using a one-way analysis of variance with a post hoc Bonferroni multiple comparison test (GraphPad Software Inc., La Jolla, CA, USA). All results were considered statistically significant at *p *< 0.01.

## Results

The MICs and MBCs of nisin, the licorice polyphenols (glabridin, licoricidin, licochalcone A), and chlorhexidine against three strains of *E. faecalis* are given in Table 1. The MICs of nisin ranged from 12.5 to 25 µg/mL. Twice these concentrations of nisin corresponded to the MBCs. The MICs of the licorice polyphenols ranged from 6.25 to 25 µg/mL, while the MBCs ranged from 50 to 200 µg/mL. In terms of MICs, licoricidin and licochalcone A were slightly more effective than glabridin. Chlorhexidine, which was used as a positive inhibitory control, had MIC and MBC values of 6.25 and 50 µg/mL, respectively.

**Table 1:** Minimum inhibitory concentration (MIC) and minimum bactericidal concentration (MBC) values of nisin, licorice polyphenols (glabridin, licoricidin, licochalcone A), and chlorhexidine against three strains of *E. faecalis*

Compound	Concentration of compound (µg/mL)		
	ATCC 19433	0DOT	1DOT
Nisin			
MIC	12.5	25	25
MBC	25	50	50
Glabridin			
MIC	25	25	25
MBC	100	50	50
Licoricidin			
MIC	6.25	12.5	6.25
MBC	50	200	100
Licochalcone A			
MIC	12.5	12.5	12.5
MBC	100	200	200
Chlorhexidine			
MIC	6.25	6.25	6.25
MBC	50	50	50

The effect of a sub-inhibitory concentration (1/4 MIC) of nisin, glabridin, licoricidin, licochalcone A, and chlorhexidine on biofilm formation by *E. faecalis* ATCC 19433 is reported in Table 2. At this concentration, none of the compounds caused a significant decrease in bacterial growth. While glabridin and licochalcone A did not attenuate biofilm formation, nisin and licoricidin caused a reduction in biofilm formation of 31.6% and 23.5%, respectively. The sub-inhibitory concentration of chlorhexidine reduced the ability of *E. faecalis* to form a biofilm by 37.5%.

**Table 2:** Effects of nisin, licorice polyphenols (glabridin, licoricidin, licochalcone A), and chlorhexidine at a sub-inhibitory concentration (1/4 MIC) on biofilm formation by *E. faecalis* ATCC 19433

Compound (µg/mL)	Relative growth (%)	Biofilm formation (%)
None	102.3 ± 2.5	101.5 ± 4.9
Nisin (3.125 µg/mL)	109.1 ± 9.9	76.5 ± 6.6*
Glabridin (6.25 µg/mL)	101.4 ± 2.9	103.8 ± 6.8
Licoricidin (1.56 µg/mL)	104.4 ± 2.2	68.4 ± 4.9*
Licochalcone A (3.125 µg/mL)	97.1 ± 0.9	91.4 ± 8.7
Chlorhexidine (1.56 µg/mL)	106.8 ± 3.7	62.5 ± 1.5*

*Significant reduction (*p *< 0.01) compared to control cultures without compounds

The synergistic antibacterial effect of nisin/glabridin, nisin/licoricidin, and nisin/licochalcone A on *E. faecalis* ATCC 19433 were assessed using the checkerboard assay. As reported in Table 3, nisin acted in synergy with all three licorice polyphenols. With an FICI of 0.09, nisin/glabridin displayed the most marked synergistic antibacterial effect.

**Table 3:** Fractional inhibitory concentration index (FICI) values for combinations of nisin and licorice polyphenols against *E. faecalis* ATCC 19433

Compound A	Compound B	FICI*	Effect
Nisin	Glabridin	0.09	Synergistic
Nisin	Licoricidin	0.25	Synergistic
Nisin	Licochalcone A	0.5	Synergistic

*FICI ≤ 0.5: synergistic effect; FICI > 0.5 and ≤ 4.0: no effect; FICI > 4.0: antagonistic effect

Given the synergistic antibacterial interactions observed with the combinations of nisin and the licorice polyphenols, the second part of the present study investigated nisin/glabridin, nisin/licoricidin, and nisin/licochalcone A, which were tested for their bactericidal effects on pre-formed *E. faecalis* biofilms. Following a 30 min contact, none of the combinations caused the desorption of the biofilm (data not shown). As reported in Table 4, nisin/licoricidin and nisin/licochalcone A resulted in > 75% biofilm killing, while nisin/glabridin resulted in 61.4% killing.

**Table 4:** Effects of nisin/glabridin, nisin/licoricidin, and nisin/licochalcone A on the killing of a pre-formed *E. faecalis* ATCC 19433 biofilm after a 30-min contact

Compound	% biofilm killing
None (PBS treatment)	0
Nisin (12.5 µg/mL) + Glabridin (25 µg/mL)	61.4 ± 6.0*
Nisin (12.5 µg/mL) + Licoricidin (6.25 µg/mL)	75.8 ± 3.1*
Nisin (12.5 µg/mL) + Licochalcone A (12.5 µg/mL)	76.4 ± 3.0*

*Significantly different (*p *< 0.01) from control biofilms treated with PBS

As the biocompatibility of antimicrobial agents used for root canal disinfection is an important aspect that must be taken into consideration, we investigated the cytotoxic effects of nisin/glabridin, nisin/licoricidin, and nisin/licochalcone A on three human cell types: oral epithelial cells, gingival fibroblasts, and stem cells of the apical papilla. As reported in Table 5, with the exception of nisin/glabridin (both compounds used at their MIC) that caused a reduction in the viability stem cells of the apical papilla, all the combinations tested exhibited low toxicity.

**Table 5:** Effects of nisin/glabridin, nisin/licoricidin, and nisin/licochalcone A on the viability of oral epithelial cells, gingival fibroblasts, and stem cells of the apical papilla (SCAP) after a 2-h contact

Mixtures	Cell viability (%)		
	Oral epithelial cells	Gingival fibroblasts	SCAP
None (control)	100	100	111.8 ± 9.5
Nisin (25 µg/mL)/Glabridin (50 µg/mL)	71.5 ± 9.2*	82.5 ± 5*	5.0 ± 2.1*
Nisin (12.5 µg/mL)/Glabridin (25 µg/mL)	115.0 ± 19.4	148.2 ± 11.0	122.4 ± 3.6
Nisin (25 µg/mL)/Licoricidin (12.5 µg/mL)	134.3 ± 2.4	116.0 ± 2.5	107.2 ± 6.0
Nisin (12.5 µg/mL)/Licoricidin (6.25 µg/mL)	131.1 ± 3.6	113.5 ± 5.4	104.2 ± 8.4
Nisin (25 µg/mL)/Licochalcone A (25 µg/mL)	139.3 ± 4.2	99.8 ± 14.1	116.9 ± 6.7
Nisin (12.5 µg/mL)/Licochalcone A (12.5 µg/mL)	137.0 ± 5.6	104.0 ± 18.3	114.4 ± 4.5

*Significant decrease (*p *< 0.01) compared to untreated control cell

**Fig. 1:**
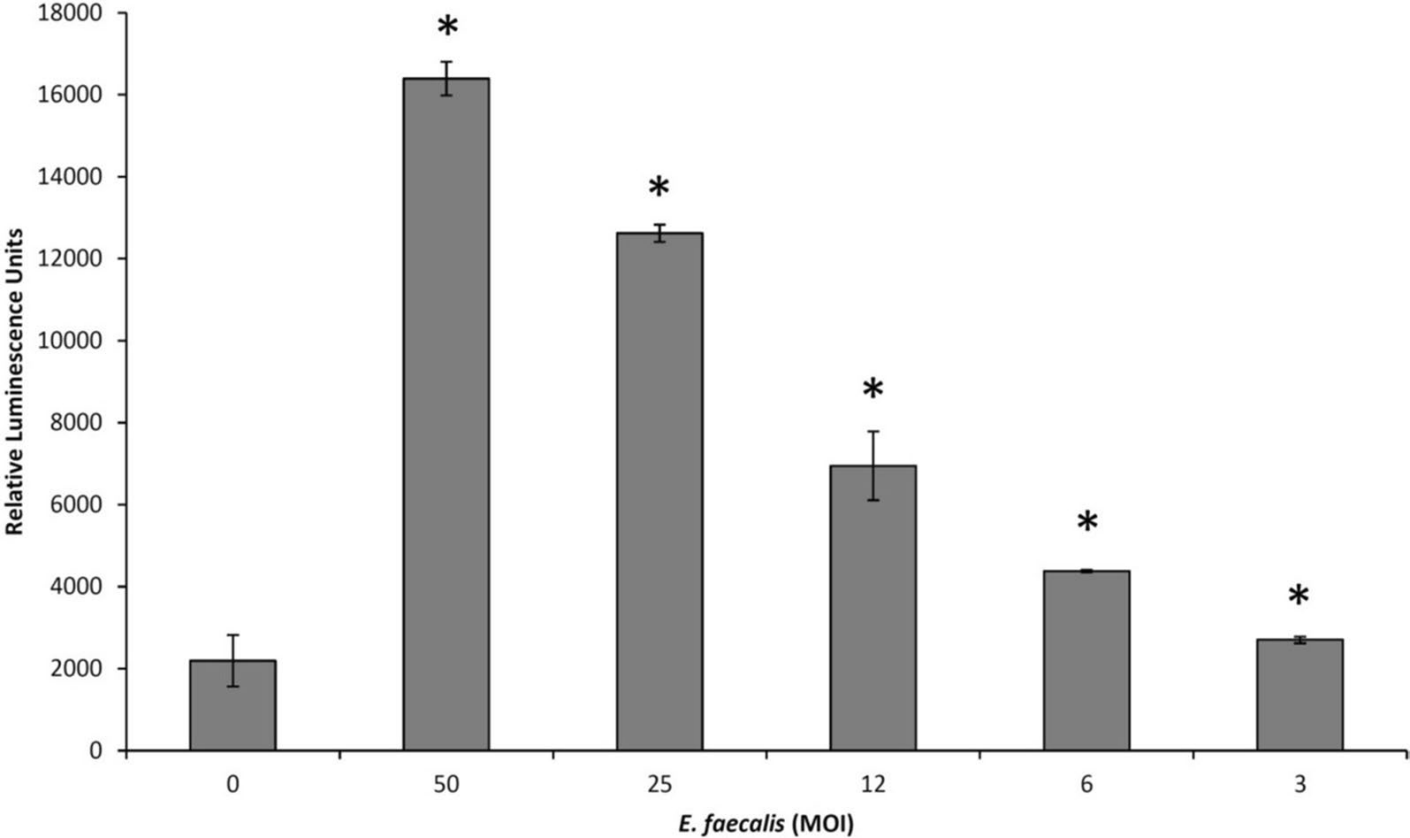
Effects of *E. faecalis* ATCC 19433 on NF-κB activation using the human monoblastic leukemia cell line U937-3xκB-LUC. *Significant increase (*p *< 0.01) compared to unstimulated control cells

**Fig. 2:**
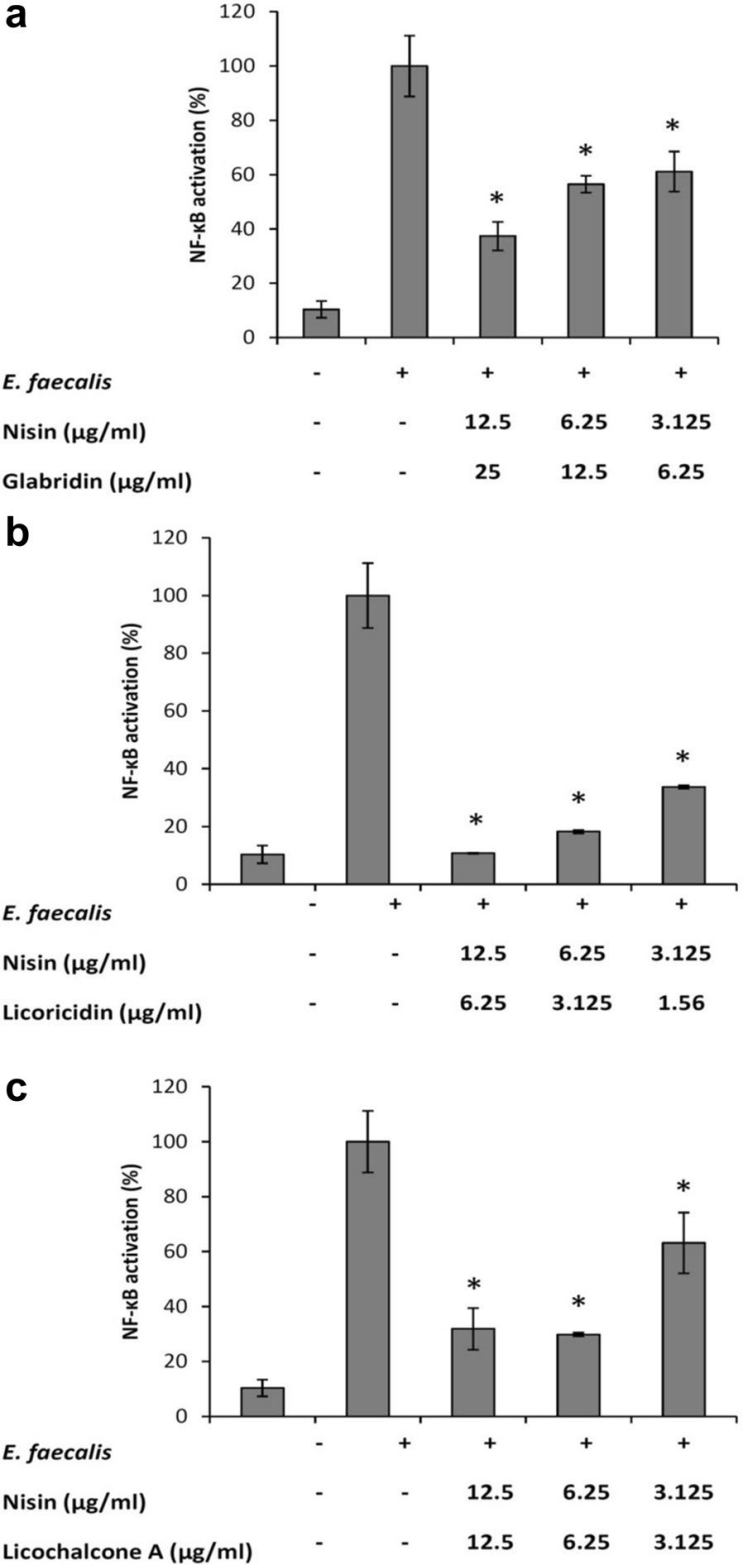
Effects of nisin/glabridin, nisin/licoricidin, and nisin/licochalcone A on *E. faecalis*-mediated NF-κB activation using the human monoblastic leukemia cell line U937-3xκB-LUC. *Significant reduction (*p *< 0.01) compared to *E. faecalis*-stimulated cells (no compounds)

Lastly, we investigated whether nisin/glabridin, nisin/licoricidin, and nisin/licochalcone A exert an anti-inflammatory effect through the inhibition of NF-κB activation using a monocyte cell line stably transfected with a luciferase gene coupled to a promoter of three NF-κB binding sites. We first showed that *E. faecalis* activates the NF-κB signaling pathway in a dose-dependent manner (Fig. 1). We observed a 7.5-fold activation of the NF-κB signaling pathway at an MOI of 50. The monocytes were pre-incubated for 30 min with the mixtures containing nisin and the licorice polyphenols at their MICs, ½ MICs, and ¼ MICs prior to stimulating the cells with *E. faecalis* at an MOI of 50. As shown in Fig. 2, all the combinations dose-dependently inhibited *E. faecalis*-mediated NF-κB activation. More specifically, when used at a concentration corresponding to ¼ MIC, nisin/glabridin, nisin/licoricidin, and nisin/licochalcone A reduced NF-κB activation by 38.9%, 66.4%, and 36.9%, respectively. No cytotoxic effects were associated with these treatments (data not shown).

## Discussion

*E. faecalis* is the bacterial species most commonly associated with failed root canal treatments and persistent root canal infections (Roças et al. 2004; Sedgley et al. 2006). This is related to its ability to colonize the dentinal tubules, form a biofilm, survive nutrient limitation, and be particularly resistant to several antimicrobial agents. *E. faecalis* resistant to root canal treatments can sustain an inflammatory response leading to chronic apical periodontitis. Although a number of intracanal medications have been proposed for the chemo-mechanical preparation of the root canal, the search for novel alternatives is still an active field of research. The aim of the present study was to evaluate the synergistic antibacterial effects of nisin and selected licorice polyphenols (glabridin, licoricidin, licochalcone A) on planktonic and biofilm-embedded *E. faecalis* cells. The biocompatibility and anti-inflammatory properties of nisin/licorice polyphenol combinations were also investigated.

Nisin A, a lantibiotic bacteriocin produced by *L. lactis* that mainly exhibits antibacterial activity against Gram-positive bacteria, is particularly relevant with regard to persistent endodontic infections involving *E. faecalis*. We showed that this antimicrobial peptide was active against *E. faecalis*, with MBCs ranging from 12.5 to 25 µg/mL. This in agreement with a previous study by Kajwadkar et al. (2017), who reported that nisin Z, which differs from nisin A by a single amino acid substitution at position 27, has antibacterial activity against *E. faecalis*. Interestingly, nisin has also been reported to be active against a number of oral pathogens associated with dental caries and periodontal diseases (Shin et al. 2015; Tong et al. 2014). We then showed that two isoflavonoids (glabridin, licoricidin) and a chalcone (licochalcone A), which are important constituents of licorice root, also efficiently inhibit the growth of *E. faecalis*, with MICs ranging from 6.25 to 25 µg/mL.

We used a broth microdilution checkerboard assay to show that combinations of nisin and licorice polyphenols exhibited significant synergistic antibacterial effects, especially nisin/glabridin. Although the exact mechanism of synergy is not understood, it may be related to the fact that nisin and the licorice polyphenols exert their antibacterial effect via different mechanisms. For instance, the membrane-permeabilizing activity of nisin (Lubelski et al. 2008) may increase the uptake of licorice polyphenols that, in turn, may have an intracellular target. Interestingly, the combinations of nisin and licorice polyphenols were also effective in the killing of pre-formed biofilms of *E. faecalis*. Further studies should evaluate theirs effects on polymicrobial biofilms, as observed in infected root canals.

Nisin is well known to be mostly active against Gram-positive bacteria (Lubelski et al. 2008), while root canal infections also involve Gram-negative bacteria. Combining this bacteriocin with licorice polyphenols allows to develop an irrigating solution with a much larger spectrum of antimicrobial activity. Indeed, Marcoux et al. (2020) recently reported that licochalcone A, licoricidin, and glabridin had MIC values ranging from 1.56 to 50 µg/mL against Gram-negative endodontic bacterial pathogens as well as *Candida albicans*.

In view of their potential use for root canal disinfection, determining the biocompatibility of the active compounds is critical. We used three different cell models (oral epithelial cells, gingival fibroblasts, and stem cells of the apical papilla) to show that the nisin/licorice polyphenol combinations had no cytotoxic effects, with the exception of nisin/glabridin, when used at their MICs. This is in agreement with a previous study by Shin et al. (2015), who reported that orally relevant human cells, including oral keratinocytes, gingival fibroblasts, periodontal ligament cells, and osteoblast-like cells, are not affected by up to 200 µg/mL of nisin.

Endodontic pathogens and the toxins they release can affect the periradicular tissues and induce periapical inflammatory lesions, which are characterized by the recruitment of inflammatory cells, including polymorphonuclear neutrophils and macrophages (Gomes and Herrera 2018). The transcription factor NF-κB of inflammatory cells is activated by a wide variety of stimuli, including bacterial pathogens, and has many target genes, including those that encode cytokines and matrix metalloproteinases that modulate periradicular tissue and bone destruction (Kumar et al. 2004). NF-κB is thus a central player in inflammatory diseases such as apical periodontitis, and the inhibition of its activation is considered a promising therapeutic strategy (Gupta et al. 2010). In the present study, we showed that nisin/glabridin, nisin/licoricidin, and nisin/licochalcone A inhibit NF-κB activation induced by *E. faecalis* in a monocyte model, suggesting that these combinations possess anti-inflammatory properties. This supports a previous study by Bodet et al. (2008), who investigated the effect of a licorice extract on the periodontopathogen-induced inflammatory response in macrophage and whole blood models and found that the extract exerted a potent anti-inflammatory effect by inhibiting the secretion of several pro-inflammatory cytokines, including IL-1β, IL-6, IL-8, and TNF-α. Moreover, licoricidin reduced inflammatory cytokine and matrix metalloproteinase secretion in a macrophage model stimulated with LPS (La et al. 2011), while licochalcone A has been reported to inhibit NF-κB activation (Furusawa et al. 2009).

In conclusion, the present study showed that combinations of nisin and glabridin, licoricidin, or licochalcone possess a number of desirable properties for root canal irrigation such as antibacterial activity, anti-inflammatory activity, and lack of cytotoxicity. Future studies should thus focus on determining whether these combinations can dissolve organic matter. Although our in vitro results were promising, clinical studies will be needed to assess their efficacy and safety.

## Data Availability

The corresponding author is responsible for providing all experimental data upon request.
